# Phytobezoar causing small bowel obstruction in a patient with Crohn's disease: A case report

**DOI:** 10.1016/j.ijscr.2022.107615

**Published:** 2022-09-08

**Authors:** Eduardo Serpa, Emmanuel Luciano, Felipe Pacheco, Wael Solh

**Affiliations:** Central Michigan University College of Medicine, Saginaw, MI 48602, United States

**Keywords:** Case report, Phytobezoar, Crohn's disease, Small bowel obstruction

## Abstract

**Introduction and importance:**

Bezoars form in any location in the gastrointestinal tract with the small bowel being uncommon. The presentation with a small bowel obstruction (SBO) is rare, representing less than 1 % of cases. Phytobezoar causing a SBO in the setting of Crohn's disease is exceedingly rare with only three cases reported in the literature.

**Case presentation:**

This case details the presentation and operative management of a phytobezoar causing small bowel obstruction in a patient with Crohn's disease. The patient is a 69-year-old male presenting with nausea, emesis, and obstipation. Imaging performed indicated a SBO with an obstructing intraluminal foreign body. The patient required exploration and a large phytobezoar was identified at the point of obstruction. This was treated with a segmental resection. The postoperative course was complicated by an anastomotic leak with re-exploration and end ileostomy.

**Clinical discussion:**

Phytobezoars are formed from indigestible plant residue which can accumulate and form a foreign body causing an obstruction in the small bowel. This is a rare occurrence in the setting of Crohn's disease. Most of these cases are managed surgically with a strictureplasty and enterotomy or a small bowel resection.

**Conclusion:**

Phytobezoars in the setting of Crohn's disease is very unusual. The pathophysiology of the disease predisposes patients to strictures and the mass-like foreign body can cause a bowel obstruction. This is typically managed surgically with a strictureplasty and enterotomy or in our case with an enterectomy.

## Highlights

• Small bowel obstruction secondary to a phytobezoar in Crohn's disease
• 4th case reported in the medical literature
• Second case to undergo surgical resection
• First case requiring Ileostomy creation

## 1 Introduction and importance

Bezoars can be formed in any area of the gastrointestinal (GI) tract, with the stomach being the most common site and infrequently in the small bowel [1]. Phytobezoars are concretions of undigested organic material in the GI tract [2]. A meta-analysis of 1061 patients who underwent diagnostic laparoscopy for acute small bowel obstruction (SBO) found a 0.8 % incidence of phytobezoars as a cause of the SBO [3]. Risk factors for phytobezoar formation include a history of a partial gastrectomy, diabetes mellitus and cystic fibrosis [4]. A phytobezoar causing a SBO in the setting of Crohn's disease (CD) appears to be very rare with only 3 cases found in the literature [5], [6], [7]. We report the details of the presentation and operative management of a case of phytobezoar causing a SBO in a patient with CD in an academic hospital. This work has been reported in line with the SCARE 2020 criteria [8].

## 2 Case presentation

**Image 1:**
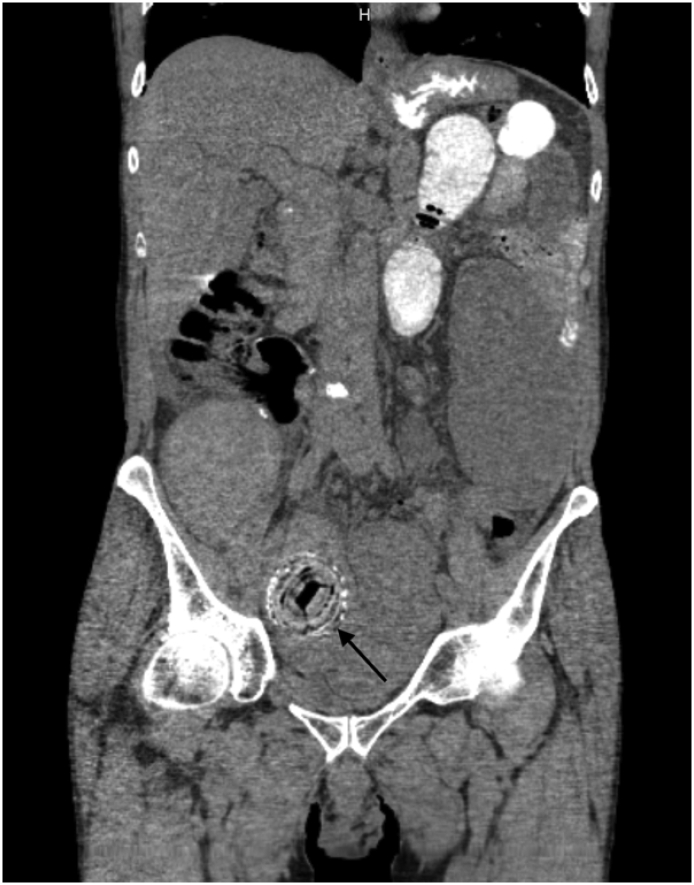
Coronal CT scan with oral contrast - dilated small bowel loops, foreign body (arrow).

**Image 2:**
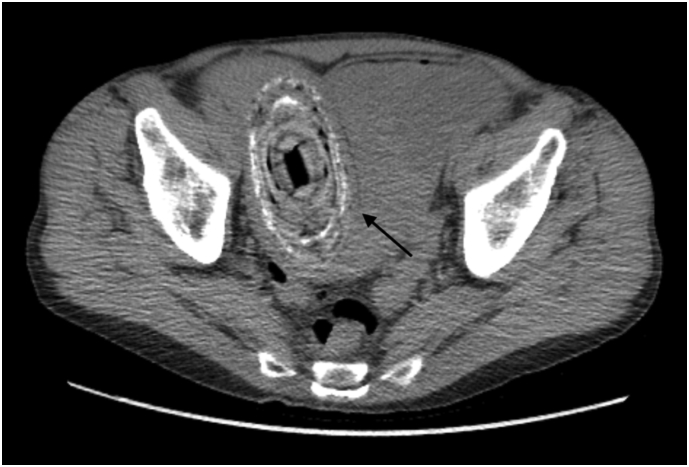
Axial CT scan - foreign body in small bowel (arrow).

The case presented is a 69-year-old male with a 40-year history of CD maintained on biologic agents and steroid therapy and a remote surgical history of ileocecectomy related to CD complications. The patient had no history of psychiatric illness, was a nonsmoker and had no history of significant alcohol abuse. He presented to the emergency department (ED) with the acute onset of diffuse abdominal pain associated with nausea and multiple episodes of emesis. The patient's last bowel movement was two days prior to presentation. The patient reported similar episodes over the past three years that were treated conservatively with steroids. On physical examination, the abdomen was distended with diffuse tenderness. Computed tomography (CT) of the abdomen and pelvis revealed dilated bowel loops and an ovoid shaped 10 cm × 5 cm × 5 cm foreign body in the small bowel (Image 1, Image 2).

**Image 3:**
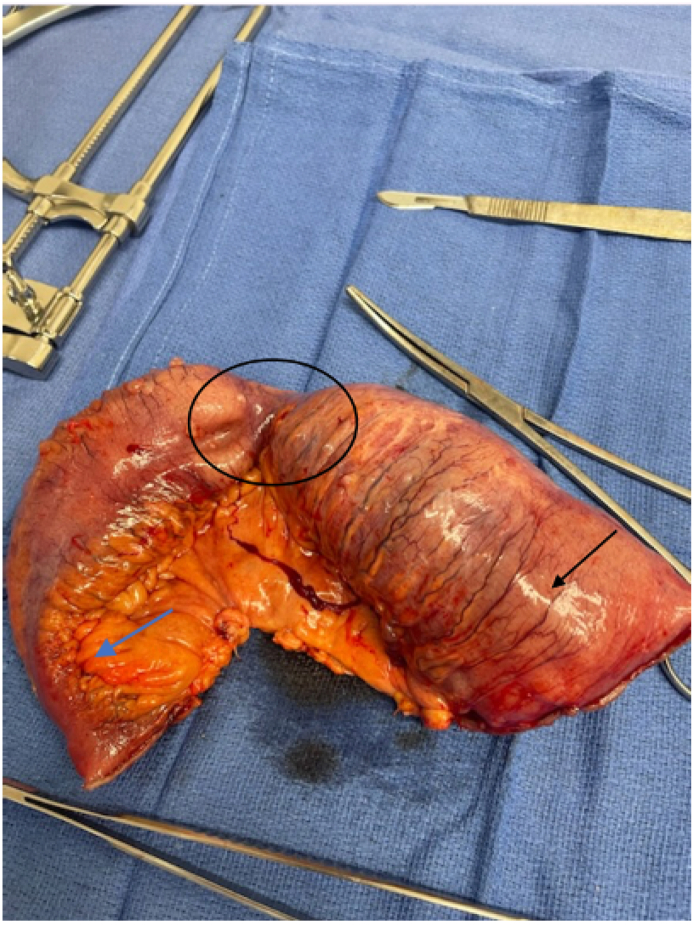
Small bowel specimen (Black Arrow: site of phytobezoar, circle: proximal stricture, blue arrow: distal stricture).

**Image 4:**
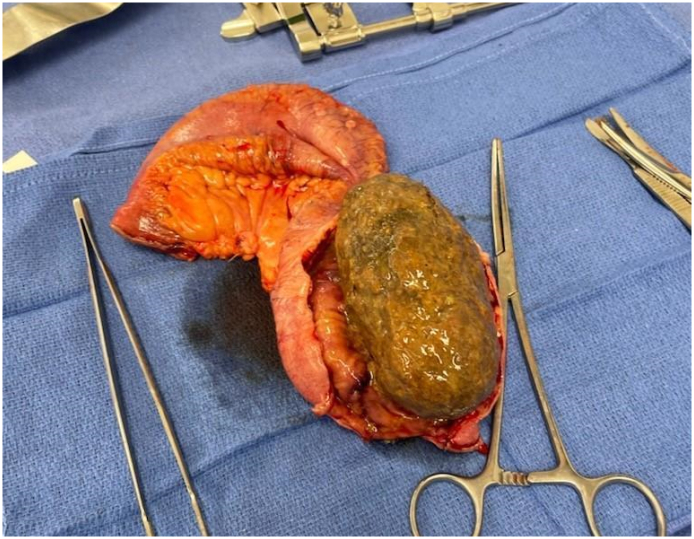
Phytobezoar.

Given the imaging evidence of an impacted foreign body with a complete SBO, an urgent abdominal exploration was indicated. The procedure was performed by the senior colorectal attending surgeon. A standard midline laparotomy was made, and the peritoneal cavity was explored. A large phytobezoar was found impacted at the site of a stricture in the small bowel approximately 160 cm proximal to the ileocolic anastomosis. The anastomosis was patent with no active inflammation. A second stricture was identified 20 cm distal to the first. No other evidence of active inflammatory bowel disease was found. A segmental small bowel resection was performed to include both strictures (Image 3). The bowel was opened on the back table and the phytobezoar examined (Image 4). A stapled side to side, entero-enterostomy anastomosis was constructed.

The postoperative course was complicated by an anastomotic leak on postoperative day 5. This required return to the operating room and resection of the anastomosis with an end ileostomy. The patient recovered well, had return of bowel function and was discharged from the hospital on postoperative day 10 from the index operation. Postoperative follow-up of the patient revealed no complications. The inflammatory bowel disease maintenance medication was continued on discharge.

## 3 Clinical discussion

Phytobezoar causing a SBO is relatively uncommon, and the management is usually surgical [2]. The phytobezoar is formed from indigestible plant residue which can accumulate and form a mass-like foreign body [9]. This can cause a mechanical obstruction in the small bowel. A phytobezoar causing a SBO in the setting of Crohn's disease is a rare event and the fourth case reported in the literature. All of these cases had an obstructing phytobezoar proximal to a stricture in the ileum and were treated with surgical intervention. In a case by Taylan et al. the management consisted of a strictureplasty and evacuation of the phytobezoar through an enterotomy [6]. In our case a different approach was taken with a small bowel resection as the second stricture was in close proximity to the first. This eliminated the need for multiple anastomoses. Similarly, to our technique, in the case reported by Harrington et al. a small bowel resection was performed due to a second stricture distal to the obstructing one [5]. Our case was complicated with an anastomotic leak requiring re-exploration with end-ileostomy creation. It is unclear in the current literature if the use of biologic agents could increase the leak rate in these types of patients.

## 4 Conclusion

Patients with obstructing phytobezoars require surgical management in the majority of cases as spontaneous resolution is unlikely. The surgical management usually requires bowel resection or strictureplasty as other reports have described. The comorbidities of the patient should be considered such as comorbid diseases, polypharmacy and poor nutritional status, potentially increasing the morbidity of the operation.

## Consent

Written informed consent was obtained from the patient for publication of this case report and accompanying images. A copy of the written consent is available for review by the Editor-in-Chief of this journal on request.

## Sources of funding

No external funding was available for this study.

## Ethical approval

None required.

## CRediT authorship contribution statement

Eduardo Serpa, MD; Conceptualization, methodology and writing original draft.

Emmanuel Luciano, MD; Conceptualization, writing original draft, review, and editing.

Felipe Pacheco, MD; writing original draft, review, and editing.

Wael Solh, MD; final review and editing.

## Research registration

N/a.

## Guarantor

Eduardo Serpa, MD.

## Provenance and peer review

Not commissioned, externally peer-reviewed.

## Declaration of competing interest

None declared.
